# Extending IGF-1 Receptor Inhibition Beyond Thyroid Eye Disease: A Scoping Review of Teprotumumab Administration in Pretibial Myxedema

**DOI:** 10.1016/j.aed.2026.04.001

**Published:** 2026-04-20

**Authors:** Maxim John Levy Barnett, Maria Helena Siqueira Tavares de Melo, Maria Luiza de Medeiros Rego

**Affiliations:** Department of Internal Medicine, Jefferson-Einstein Hospital, Philadelphia, Pennsylvania

## Abstract

**Background:**

Pretibial myxedema is an infrequent dermatologic manifestation of autoimmune thyroid disease characterized by glycosaminoglycan deposition within the dermis. Treatment options are limited, lacking durable effect and remain largely palliative. Emerging evidence, however, suggests pretibial myxedema may share a common pathophysiology with thyroid eye disease, raising the possibility that inhibition of the insulin-like growth factor-1 receptor may offer therapeutic benefit.

**Methods:**

With the scant literature documenting a response (or lack thereof) to teprotumumab therapy for pretibial myxedema, our objective is to thematically analyze and review the existing literature pertaining to this lesser-known phenomenon, with particular emphasis on clinical response and durability. A scoping review was performed with a search of 3 databases between January 2020 and 2026. Eligible studies included patients with pretibial myxedema with exposure to teprotumumab. Data extraction included study characteristics, patient demographics, treatment regimens, time to clinical improvement, relapse patterns, and follow-up duration.

**Results:**

Overall, *n* = 14 studies met inclusion criteria (*n* = 31 patients). All studies consistently noted an improvement with clinical responses noted early, often within the first to fourth infusions. Relapses occurred in a subset of patients, often months after completing treatment; however, repeat exposure continued to demonstrate persistent benefits in many reported studies.

**Conclusions:**

Teprotumumab demonstrated improvement in pretibial myxedema, supporting the notion of a shared pathophysiologic basis with thyroid eye disease. Prospective registries and standardized outcome measures are needed to define durability, retreatment strategies, and long-term safety.

**Highlights**

• Pretibial myxedema is a rare dermatologic manifestation of Graves’ disease
• Evidence-based therapies for the management of pretibial myxedema are limited
• Pretibial myxedema mirrors the pathophysiology of thyroid eye disease
• Limited evidence suggests teprotumumab is efficacious in pretibial myxedema

**Clinical Relevance**

While pretibial myxedema is an infrequent manifestation of Graves’ disease, it can lead to significant functional impairment, and a reduced quality of life. Although no therapies are currently approved for treatment, emerging evidence suggests teprotumumab may be effective, supported by shared underlying pathophysiology with thyroid eye disease.

## Introduction

Pretibial myxedema (PTM) is an infrequent dermatologic manifestation of autoimmune thyroid disease, characterized by excess accumulation of glycosaminoglycans (particularly hyaluronic acid) within the dermis and subcutaneous tissues, resulting in nonpitting edema, skin thickening, plaque, or nodular changes, in a bilateral, asymmetric distribution.^1^ Hyperthyroidism (Graves’ disease) accounts for more than 90% of cases of PTM, although it has been described among euthyroid and hypothyroid patients.^2^ PTM was first described by American pathologist Ludvig Hektoen in 1895, and has subsequently been referred to as thyroid dermopathy, localized myxedema, myxedema tuberosum, and Jadassohn–Dösseker syndrome.^3^^,^^4^

PTM forms a component of the classic triad of extrathyroidal manifestations of Graves’ disease, alongside thyroid eye disease (TED) and acropachy; however, fulfilment of the complete triad is uncommon (occurring in less than 1% of patients).^5^ Between 25% and 50% of patients with Graves’ disease will have TED, compared to PTM in 4% (and acropachy 0.3%), however, the presence of TED significantly increases the risk of PTM by up to 15%.^2^^,^^6^, ^7^, ^8^ Intriguingly, acropachy occurs in 20% of patients with PTM and does not present in the absence of dermopathy.^9^^,^^10^

PTM most often develops within 12-24 months following the diagnosis of thyrotoxicosis, with ophthalmopathy preceding dermopathy in more than 75% of cases.^5^^,^^11^ There appears to be a female preponderance (female-to-male ratio 3.5:1), with a peak incidence between the fifth and sixth decades.^12^^,^^13^ Four predominant variants of PTM have been described within the literature: nonpitting edema (43.3%), plaque-type (27%), nodular (18.5%) and elephantiasic (2.8%).^2^ Along with morphologic heterogeneity, PTM progresses through 4 clinical stages: active, stable, sclerotic and recession, with patients at risk for oscillating between stages of disease without treatment.^3^ While often asymptomatic, severe forms may lead to discomfort, functional impairment and cosmetic disfiguration.^14^ In 99% of cases, the pretibial region is involved, which likely reflects a combination of the gravitational venous stasis (with pooling of inflammatory mediators) and local trauma, however, other sites such as the feet, toes, upper extremities, face, and trunk have been described.^2^^,^^9^

Early identification and treatment of PTM is vital, as delays limit the effectiveness of therapy.^15^ While mild PTM often requires no treatment (with 50% likely to achieve spontaneous remission over years), severe cases necessitate aggressive therapy.^14^ At present, there are no Food and Drug Administration approved treatments for PTM, and management is not specifically addressed in major endocrine guidelines. Of the available options (Table 1), these are largely palliative and lack durable effects, with recurrence being a common issue.^14^^,^^16^

**Table 1:** Available Treatment Options

Category	Treatment
Corticosteroid therapy	Topical steroids, intralesional steroids, systemic steroids
Mechanical therapy	Compression therapy, pneumatic pump therapy
Immunomodulatory therapy	Rituximab, intravenous immunoglobulin, plasmapheresis
Other therapies	Octreotide, hyaluronidase, pentoxifylline
Phototherapy	Ultraviolet A1 phototherapy
Surgical options	Surgical excision, incision, and drainage

Rather than a localized cutaneous disorder, increasing evidence suggests a parallel process to the pathogenesis of TED.^16^ This shared pathophysiology suggests a systemic process and involves thyroid-stimulating hormone receptor (TSHR) antibodies activating fibroblasts through direct engagement and through crosstalk with insulin-like-growth factor-1 receptors (IGF-1R), promoting cytokine infiltration and glycosaminoglycan accumulation.^16^ Biopsies of patients with PTM have confirmed the presence of such receptors, confirming a shared mechanistic pathway.^17^ Risk factors common to both conditions include smoking, radioiodine ablation, and elevated titers of TSHR antibodies.^18^^,^^19^

Teprotumumab is currently the only FDA-approved medical therapy for TED, first approved in 2020.^20^ Teprotumumab is a human IgG_1_ monoclonal antibody inhibiting IGF-1R–mediated signaling and disrupting its interaction with TSHR antibodies, preventing the fibroblast activation pathway central to TED pathogenesis.^21^ It is administered as a loading dose of 10 mg/kg, followed by 20 mg/kg every 3 weeks for the remaining 7 infusions (totalling 6 months of treatment).^22^ With the accumulating evidence of a shared fibroinflammatory mechanism among TED and PTM, it is biologically plausible that the benefits of teprotumumab may extend to dermopathy (and acropachy). Indeed, a growing body of case-based literature has reported improvement of PTM in patients receiving teprotumumab for TED.^16^ However, these data remain fragmented, and long-term outcomes are incompletely characterized.

The purpose of this scoping review is therefore to systematically examine the existing literature on teprotumumab use in PTM, perform a thematic analysis of reported outcomes, and identify key themes related to treatment response, durability, and mechanistic rationale.

## Methods

### Protocol and Framework

This scoping review was conducted in accordance with the Joanna Briggs Institute methodology for scoping reviews.^23^ Reporting followed the Preferred Reporting Items for Systematic Reviews and Meta-Analyses extension for Scoping Reviews to ensure transparency and methodological rigor.^24^ The review was guided by the following research question: “What evidence exists regarding the use of teprotumumab as a treatment modality for PTM in patients with autoimmune thyroid disease?”

### Eligibility Criteria

A Population-Concept-Context framework was used to define eligibility. Our population was defined as patients with autoimmune thyroid disease without restrictions on age or sex. Our concept included use of teprotumumab, regardless of dosing duration or treatment completion. The context included all clinical settings (inpatient or outpatient), without geographic or language restrictions. Studies were eligible if they reported clinical outcomes of PTM in patients treated with teprotumumab. Conference proceedings, case reports, case series, observational studies, and randomized controlled trials were included. Review articles without original patient data were excluded unless they presented new case-level information. Although thyroid acropachy was not an explicit component of the search strategy, cases in which acropachy co-occurred with PTM were included when reported within eligible studies.

### Search Strategy

A comprehensive literature search was performed across 3 databases (PubMED/MEDLINE, CINAHL, and Google Scholar), identifying relevant articles relating to teprotumumab and pretibial myxedema between January 2020 and January 2026. Reference lists of included studies were manually screened to identify additional relevant publications.

### Study Selection

A comprehensive search across 3 databases identified a total of *n* = 258 articles. All articles were added to Microsoft excel; whereby *n* = 18 duplicates were removed. The tile and abstract were manually screened by 2 authors; when disagreement ensued, the senior author was included to decide and reach a majority, with *n* = 223, removed. This led us to *n* = 17 articles, however, *n* = 3 were initial conference/abstract poster presentations (which were subsequently turned into manuscripts and therefore presented data twice) which were excluded. Full-text screening of the remaining *n* = 14 studies was conducted, and all papers met the inclusion criteria (Figure).^25^, ^26^, ^27^, ^28^, ^29^, ^30^, ^31^, ^32^, ^33^, ^34^, ^35^, ^36^, ^37^, ^38^

**Figure:**
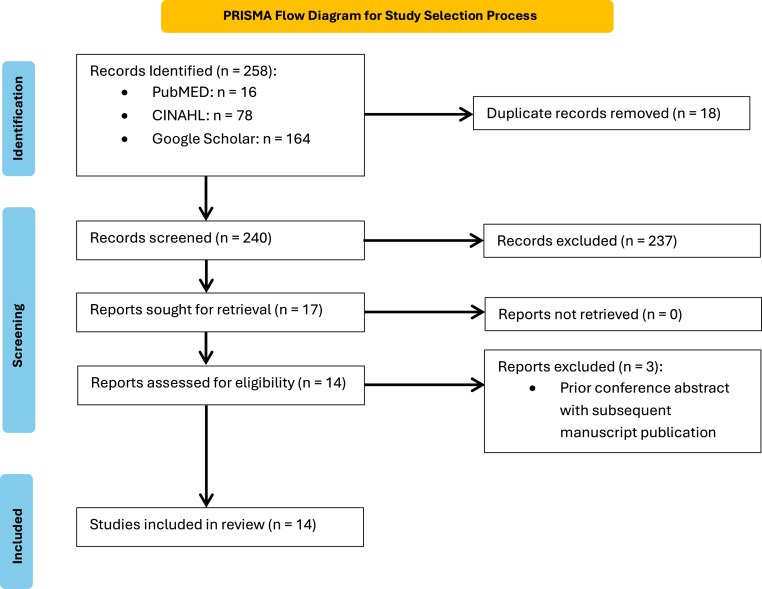
Preferred Reporting Items for Systematic Reviews and Meta-Analyses extension for scoping reviews (PRISMA-ScR) flow diagram (for study selection).

### Data Extraction and Analysis

Data were extracted using a standardized table that captured publication details (authors, year), study design, number of participants (including age, gender and ethnicity), presence of extrathyroidal manifestations, number of teprotumumab infusions, time to clinical improvement, follow-up duration, relapse, prior treatment failure, and phenotype. Extracted data were synthesized descriptively and narratively. A thematic analysis was used to organize and identify patterns related to the treatment of teprotumumab and PTM (Tables 2 and 3). In this descriptive study, categorical variables are expressed as percentages, means and ranges.

**Table 2:** Study Characteristics and Patient Demographics

Author	Year	Study design	Patients (*n*)	Age and sex	Ethnicity	PTM phenotype	Extrathyroidal manifestations
Varma et al^25^	2020	Case Report	1	49 F	Caucasian	Nodular	TED + PTM
Petit et al^26^	2021	Case Report	1	50 F	Caucasian	Plaque	TED + PTM + ACPY
Bowens et al^27^	2022	Case Report	1	51 F	African American	Plaque	TED + PTM
Emge et al^28^	2022	Case Report	1	71 F	Not reported	Not reported	TED + PTM
Crespo-Trevino et al^29^	2022	Case Series	4	Patient 1: 66 M Patient 2: 75 F Patient 3:79 M Patient 4: 52 F	Patient 1: Caucasian Patient 2: Caucasian Patient 3: African American Patient 4: Caucasian	Patient 1: Nodular Patient 2: Non-pitting edema Patient 3: Non-pitting edema Patient 4: Not reported	TED + PTM
Washington et al^30^	2022	Case Series	2	Patient 1: 42 F Patient 2: 55 M	Patient 1: Caucasian Patient 2: Caucasian	Patient 1: Plaque Patient 2: Elephantiasic	TED + PTM
Mervis et al^31^	2022	Retrospective Study	10	Not reported	Not reported	Not reported	TED + PTM
Reyes et al^32^	2023	Case Report	1	71 F	Caucasian	Nodular	TED + PTM
Patel et al^33^	2023	Case Report	1	76 F	Caucasian	Plaque	TED + PTM + ACPY
Kelly & Turbin^34^	2024	Case Report	1	66 F	Caucasian	Nonpitting edema	TED + PTM
Niedzialkowska et al^35^	2024	Case Report	1	52 F	Caucasian	Nodular (with non-pitting edema)	TED + PTM
Lira et al^36^	2025	Case Report	1	25 M	Asian	Plaque (with non-pitting edema)	TED + PTM + ACPY
Chatterjee^37^	2025	Case Report	1	49 F	Caucasian	Non-pitting edema	TED + PTM + ACPY
Hamadi et al^38^	2026	Case Series	5	Patient 1: 83 F Patient 2: 66 M Patient 3: 71 M Patient 4: 78 F Patient 5: 71 F	Patient 1: Caucasian Patient 2: Caucasian Patient 3: Caucasian Patient 4: Caucasian Patient 5: Caucasian	Patient 1: Nodular Patient 2: Elephantiasic Patient 3: Nodular Patient 4: Plaque Patient 5: Plaque	TED + PTM

Abbreviations: ACPY, acropachy; F, female; M, male; PTM, pretibial myxedema; TED, thyroid eye disease.

**Table 3:** Treatment Regimen and Clinical Outcomes

Author	Year	Infusions	Time to improvement	Follow-up	Relapse	Prior treatment failure
Varma et al^25^	2020	7	After 2nd infusion	4.5 mo	No	Yes
Petit et al^26^	2021	8	After 1st infusion	5 mo	Yes	Yes
Bowens et al^27^	2022	8	Not reported	Not reported	Not reported	Not reported
Emge et al^28^	2022	8	Not reported	6 mo	Yes	Yes
Crespo-Trevino et al^29^	2022	Patient 1: 8 Patient 2: 8 Patient 3: 8 Patient 4: 8	Patient 1: After 3^rd^ infusion Patient 2: After 2^nd^ infusion Patient 3: After 2^nd^ infusion Patient 4: Not reported	Patient 1: 12 mo Patient 2: 12 mo Patient 3: 2 mo Patient 4: 12 mo	Patient 1: Yes Patient 2: No Patient 3: No Patient 4: No	Patient 1: Yes Patient 2: Yes Patient 3: Yes Patient 4: Yes
Washington et al^30^	2022	Patient 1: 5 Patient 2: 8	Patient 1: After 1^st^ infusion Patient 2: Not reported	Patient 1: 3 mo Patient 2: 6 mo	Patient 1: No Patient 2: No	Patient 1: Yes Patient 2: Yes
Mervis et al^31^	2022	Patient 1: 8 Patient 2: 8 Patient 3: 4 Patient 4: 3 Patients 5-10: Not reported	Not reported	Not reported	Not reported	Not reported
Reyes et al^32^	2023	8	Not reported (“Midway Point” which equates to 4 infusions)	7 mo	Yes	Yes
Patel et al^33^	2023	8	After 4th infusion	18 mo	No	Not reported
Kelly & Turbin^34^	2024	8	After 2nd infusion	13 mo	No	Yes
Niedzialkowska et al^35^	2024	6	After 4th infusion	17 mo	Yes	Yes
Lira et al^36^	2025	8	After 5th infusion	Not reported	Not reported	Yes
Chatterjee^37^	2025	8	After 1st infusion for acropachy (not defined to PTM)	Not reported	Not reported	Yes
Hamadi et al^38^	2026	Patient 1: - 1^st^ Course: 8 - 2^nd^ Course: 1 Patient 2: - 1^st^ Course: 8 - 2^nd^ Course: 4 - 3^rd^ Course: 5 Patient 3: 8 Patient 4: 8 Patient 5: 8	Patient 1: - 1^st^ Course: After 4^th^ infusion - 2^nd^ Course: After 1^st^ infusion Patient 2: - 1^st^ Course: Not reported - 2^nd^ Course: After 2^nd^ infusion - 3^rd^ Course: After 4^th^ infusion Patient 3: After 4^th^ infusion Patient 4: Not reported Patient 5: Not reported	Patient 1: 19 mo Patient 2: 18, 14 mo Patient 3: 4 y Patient 4: 24 mo Patient 5: 24 mo	Patient 1: Yes Patient 2: Yes Patient 3: No Patient 4: Yes Patient 5: No	Patient 1: Yes Patient 2: Yes Patient 3: Yes Patient 4: Yes Patient 5: Yes

Abbreviations: ACPY, acropachy; PTM, pretibial myxedema; TED, thyroid eye disease.

Because no validated disease-severity instruments are available for PTM, definitions of treatment response vary across studies. For the purpose of this scoping review, clinical improvement was defined as any author-reported reduction in lesion size, edema, dermal thickening, and nodularity of associated symptoms as documented through clinical examination, photographic comparison, or patient-reported functional improvement. Conversely, relapse was defined as recurrence or worsening of PTM following an initial period of improvement after teprotumumab therapy, as explicitly reported by the original study authors. When quantitative measures were unavailable, classification relied on narrative descriptions provided within the source reports.

### Quality Appraisal

Although formal quality appraisal is not mandatory for scoping reviews, an assessment of methodological rigor was conducted due to the clinical nature of the topic and the predominance of case-based evidence. Study quality was evaluated using the Joanna Briggs Institute Critical Appraisal Checklists for case reports, case series and observational studies. Each study was independently assessed across domains including diagnostic clarity, intervention description, outcome reporting, and adequacy of follow-up. Disagreements were resolved through discussion and consensus. Results of the appraisal are presented in Supplementary Table 1.

## Results

A total of *n* = 14 studies met inclusion criteria, encompassing *n* = 31 patients with PTM treated with teprotumumab. These included studies consisted of *n* = 10 case reports (*n* = 10 patients), *n* = 3 case series (*n* = 11 patients), and *n* = 1 retrospective study (*n* = 10 patients).^25^, ^26^, ^27^, ^28^, ^29^, ^30^, ^31^, ^32^, ^33^, ^34^, ^35^, ^36^, ^37^, ^38^ All studies were published between 2020 and 2026, corresponding to the post-approval period of teprotumumab. Notably, TED was present in all *n* = 31 patients (100%), as teprotumumab is currently approved exclusively for this purpose; in each study, teprotumumab was initiated for TED, with outcomes related to PTM reported concurrently.

### Clinical Response

All *n* = 14 studies reported clinical improvement in PTM following teprotumumab therapy, corresponding to improvements in *n* = 31 patients (100%).^25^, ^26^, ^27^, ^28^, ^29^, ^30^, ^31^, ^32^, ^33^, ^34^, ^35^, ^36^, ^37^, ^38^ Prior failure of therapy for PTM before teprotumumab was documented in *n* = 19 patients (61.3%).^25^^,^^26^^,^^28^, ^29^, ^30^^,^^32^^,^^34^, ^35^, ^36^, ^37^, ^38^ Time to improvement is explicitly reported in *n* = 9 studies.^25^^,^^26^^,^^29^^,^^30^^,^^33^, ^34^, ^35^, ^36^^,^^38^ Improvement after 1-2 infusions was reported in *n* = 6 studies, whereas improvement after 3-4 infusions was reported in *n* = 3 studies.^25^^,^^26^^,^^29^^,^^30^^,^^33^, ^34^, ^35^, ^36^^,^^38^ The earliest reported improvement occurred after the first infusion.^26^^,^^30^ Across studies reporting timing, most patients demonstrated visible or functional improvement by approximately the fourth infusion. Several patients did not complete the standard eight-infusion regimen due to adverse effects or access limitations; however, improvement was still reported in these cases.^30^^,^^35^

Among the patient level, infusion to response is reported in *n* = 14 patients; improvements were documented as follows: after 1 infusion (*n* = 3 patients), 2 infusions (*n* = 4 patients), 3 infusions (*n* = 1 patient), 4 infusions (*n* = 5 patients) and 5 infusions (*n* = 1 patient).^25^^,^^26^^,^^29^^,^^30^^,^^33^, ^34^, ^35^, ^36^^,^^38^ This correlated to a reported improvement after a mean of 2.8 infusions (range of 1-5 infusions). Number of infusions were reported for *n* = 25 patients, with *n* = 20 completing all 8 infusions.^25^, ^26^, ^27^, ^28^, ^29^, ^30^, ^31^, ^32^, ^33^, ^34^, ^35^, ^36^, ^37^, ^38^ Early discontinuation is reported for *n* = 2 patients, occurring due to hyperglycemia and lack of insurance coverage.^30^^,^^35^ Of the patients who underwent early discontinuation, the minimum number of infusions associated with an improvement was one infusion, while the maximum number before early discontinuation noting a positive response was after the fourth infusion.^30^^,^^35^

### Recurrence and Retreatment

Follow-up was reported in *n* = 19 patients, with durations ranging from 3 months to 4 years, with a mean follow up of 14.6 month (range of 2-48 months).^25^^,^^26^^,^^28^, ^29^, ^30^, ^31^, ^32^, ^33^, ^34^, ^35^^,^^38^ Follow up < 3 months is reported with *n* = 2 patients, 3 - <6 months *n* = 2, 6 - <12 months *n* = 4, 12 - < 18 months *n* = 5, 18 - < 24 months *n* = 5, and ≥ 24 months *n* = 1. Of these patients who underwent follow up, *n* = 9 (*n* = 6 objective and *n* = 3 subjective) demonstrated relapse (47%).^26^^,^^28^^,^^29^^,^^31^^,^^32^^,^^35^^,^^38^ Relapse occurred irrespective of incomplete or full-course (8) infusions. Moreover, early clinical improvement (within 1 - 4 infusions) was noted in both relapsing and durable cases. It is important to note that even in patients who did relapse, it was often partial and remained improved relative to baseline.^38^ Relapse occurred between 5 and 24 months after completion of teprotumumab, with a mean time to relapse of 12.1 months. Of the *n* = 11 patients with >12 months follow up, *n* = 7 patients were documented to relapse (64%). Conversely, of the *n* = 8 patients with follow up < 12 months, *n* = 2 relapsed (25%).^25^^,^^26^^,^^28^, ^29^, ^30^, ^31^, ^32^ In the case series posed by Hamadi et al^38^ (*n* = 5), after completing all 8 infusions, 2 patients are noted to relapse and undergo second (and third) infusion courses; however, these were discontinued early due to severe hearing impairment (*n* = 1), and gastrointestinal distress requiring hospitalization (*n* = 1). Both patients continued to demonstrate renewed clinical and functional improvement with second (and in one case, third) courses, with time to improvement (upon retreatment) within the first 4 infusions.

### Thyroid Function Status

Thyroid function status was reported for *n* = 13 studies (*n* = 21 patients). Irrespective of the underlying thyroid disease, all patients demonstrated clinical improvement.^25^, ^26^, ^27^, ^28^, ^29^, ^30^^,^^32^, ^33^, ^34^, ^35^, ^36^, ^37^, ^38^ Graves’ disease was present in *n* = 19 patients, with *n* = 2 harboring Hashimoto’s thyroiditis.^25^, ^26^, ^27^, ^28^, ^29^, ^30^^,^^32^, ^33^, ^34^, ^35^, ^36^, ^37^, ^38^ Among patients with Graves’ disease who were hypothyroid (with prior radioiodine ablation and/or thyroidectomy) at presentation (*n* = 14), disease severity was greatest, encompassing all nodular and elephantiasic phenotypes.^25^^,^^27^^,^^29^^,^^30^^,^^32^^,^^33^^,^^36^, ^37^, ^38^ In this subgroup, time to improvement varied by phenotype, with nonpitting and plaque-type disease improving after the first-to-second infusions, whereas nodular and elephantiasic disease typically improved after the third-to-fourth infusions or later. Most patients completed an eight-infusion course and repeat treatment courses were reported exclusively in this group. Relapse occurred in 6 patients, predominantly those with nodular or elephantiasic disease, typically between 12 and 19 months after treatment completion. In contrast, patients with Graves’ disease who were hyperthyroid at presentation (*n* = 5) predominantly exhibited nonpitting edematous or plaque-type disease, demonstrated early improvement after the first-to-second infusions, and rarely relapsed, with only one relapse reported at approximately 5–6 months.^26^^,^^28^, ^29^, ^30^ Patients with non-Graves autoimmune thyroid disease (Hashimoto; *n* = 2) exhibited only nonpitting or plaque-predominant phenotypes, demonstrated early response to therapy, completed standard eight-infusion courses, and did not experience documented relapse during follow-up.^34^^,^^35^

### Phenotype

All *n* = 31 patients (100%) had lower extremity involvement (with variations in morphologic subtype and anatomical distribution).^25^, ^26^, ^27^, ^28^, ^29^, ^30^, ^31^, ^32^, ^33^, ^34^, ^35^, ^36^, ^37^, ^38^ Across the *n* = 14 studies, the cutaneous phenotype was depictable among *n* = 19 patients, with non-pitting edema the predominant phenotype for *n* = 4, plaque (*n* = 7), nodular (*n* = 6), and elephantiasic (*n* = 2).^25^, ^26^, ^27^^,^^30^^,^^32^, ^33^, ^34^, ^35^, ^36^, ^37^, ^38^ Failure of prior treatment was most consistently reported among patients with nodular and elephantiasic PTM, who frequently had persistent disease despite multiple modalities. In contrast, patients with non-pitting edematous and plaque-type disease most commonly failed topical or intralesional corticosteroids and compression therapy alone, with fewer reports of escalation to systemic immunomodulatory treatments before teprotumumab initiation.

Among patients with nonpitting edematous PTM, most completed a standard eight-infusion course, with improvement typically noted after the first-to-second infusions and durable responses reported at 12–24 months, with relapse rarely described.^29^^,^^34^^,^^37^ Patients with plaque-type disease likewise commonly received 8 infusions (range 5–8), with early improvement frequently observed after the first-to-second infusions; however, relapse was reported in several cases, occurring as early as 5–6 months post-treatment.^26^^,^^27^^,^^30^^,^^33^^,^^36^^,^^38^ In contrast, nodular PTM demonstrated a slower response, with improvement most often reported after the third-to-fourth infusions, and a higher propensity for relapse, typically occurring between 12 and 19 months following treatment completion; several nodular cases required repeat courses of therapy.^25^^,^^29^^,^^32^^,^^35^^,^^38^ Data on elephantiasic disease were limited, albeit patients uniformly received 8 infusions, and exhibited the latest time to improvement (≥fourth infusion).^30^^,^^38^ Overall, earlier response and greater durability were observed in nonpitting and plaque phenotypes, whereas nodular and elephantiasic forms were associated with delayed response, increased relapse risk, and greater treatment intensity.

Thyroid acropachy was identified in *n* = 4 patients, representing the most severe end of the extrathyroidal disease spectrum, and uniformly coexisting with PTM and TED.^26^^,^^33^^,^^36^^,^^37^ All acropachy patients had failed multiple prior therapies but nonetheless demonstrated clinical improvement with teprotumumab, with all *n* = 4 patients completing the full 8 infusions. Time to response, however, was only documented for *n* = 1 study (after the first infusion).^37^

### Age, Gender, and Ethnicity

Exact patient age was reported among *n* = 21 patients (68%), with a mean age of 61.8 years (range 25-83 years).^25^, ^26^, ^27^, ^28^, ^29^, ^30^^,^^32^, ^33^, ^34^, ^35^, ^36^, ^37^, ^38^ Gender was reported for *n* = 25 patients (80.6%); of these, *n* = 17 female (68.0%) and *n* = 8 male (32.0%).^25^, ^26^, ^27^, ^28^, ^29^, ^30^^,^^32^, ^33^, ^34^, ^35^, ^36^, ^37^, ^38^ Moreover, ethnicity was available for *n* = 20 patients (65%), with *n* = 17 Caucasian (55%), *n* = 1 Asian (3%), *n* = 2 African American (6%), and *n* = 11 unknown (35%).^25^, ^26^, ^27^, ^28^, ^29^, ^30^^,^^32^, ^33^, ^34^, ^35^, ^36^, ^37^, ^38^

Patients younger than 50 years constituted a minority of cases and were predominantly female, with disease most often manifesting as nonpitting edema or plaque-type PTM; nodular disease was uncommon and elephantiasic disease was not observed in this age group.^25^^,^^30^^,^^36^^,^^37^ Prior to teprotumumab initiation, these younger patients most frequently had failed conservative therapies with limited escalation to systemic immunomodulatory agents or surgical interventions. Most completed a standard eight-infusion course, although abbreviated regimens of five-to-six infusions were occasionally reported due to adverse effects, and clinical improvement was typically noted early, most often after the first or second infusion. Relapse was infrequently reported in patients younger than 50 years, with sustained improvement described during available follow-up.

In contrast, patients aged 50 years or older represented the majority of reported cases and exhibited greater clinical heterogeneity, including a broader distribution of phenotypes, with nodular and elephantiasic forms occurring almost exclusively in this age group.^26^, ^27^, ^28^, ^29^, ^30^^,^^32^, ^33^, ^34^, ^35^^,^^38^ These patients more commonly demonstrated extensive treatment refractoriness prior to teprotumumab. Most completed a full eight-infusion course, although relapse was more frequently reported in this older age group. Moreover, repeat courses were reported almost exclusively among older patients, particularly those with nodular or elephantiasic disease.^38^ Time to response was more variable in this group, with plaque and nonpitting phenotypes often improving after the first-to-second infusions, whereas nodular and elephantiasic disease typically showed improvement after the third-to-fourth infusion or later. Gender distribution was more balanced among patients aged 50 years or older, with a higher proportion of male patients compared with the younger cohort.

## Discussion

PTM remains an infrequent and poorly understood extrathyroidal manifestation of Graves’ disease, and remains therapeutically challenging. Historically, PTM was regarded as a largely cosmetic complication of Graves’ disease and conceptualized as a localized cutaneous process. Consequently, treatment strategies have focused on local or symptomatic management, with limited and often transient efficacy. From a disease perspective, PTM is characterized by excessive dermal glycosaminoglycan deposition by fibroblasts, resulting in nonpitting edema, plaques, nodules, and, in severe cases, elephantiasic changes.^38^ Although these histopathologic features have long been recognized, the upstream driver of fibroblast activation in PTM was historically inferred rather than directly demonstrated. In 1997, Stadlmayr et al demonstrated fibroblasts from orbital and pretibial tissues of patients with TED and PTM expressed functional TSHR RNA and protein, supporting the idea of a shared autoantigen.^39^ Moreover, in 2024, Walsh et al provided direct histopathologic evidence supporting a shared pathophysiology between PTM and TED in a controlled immunohistochemical study, demonstrating significant upregulation of IGF-1R expression in PTM lesions compared with controls.^17^ The clinical observations summarized in this scoping review reinforce this mechanistic alignment. All included studies reported improvement in PTM following systemic administration of teprotumumab for TED, without direct targeting of skin lesions. Such a response would be difficult to explain if PTM represented a fixed structural sequela or purely localized inflammatory condition.

Our findings demonstrate a phenotype-dependent gradient of response, with earlier improvement observed in edematous disease and progressively later response in progressive phenotypes. A parallel gradient in durability was also observed, with patients with severe disease appearing to be at higher risk for relapse. The frequent observation of early clinical improvement has significance beyond convenience. Although many patients did not complete all 8 infusions for various reasons, meaningful improvement was still observed in patients receiving abbreviated courses, indicating that clinical benefit may not be dependent on treatment completion.

Relapses occurring months after treatment should not be interpreted as therapeutic failure. Rather, relapse patterns resemble those seen in TED and likely reflect re-emergence of autoimmune signaling as pharmacologic suppression wanes. All patients in this review who underwent retreatment experienced renewed clinical improvement, reinforcing the interpretation of PTM as a chronic, relapsing immune-mediated condition rather than a static dermatologic sequela. Predictors of relapse and optimal retreatment protocols; however, remain undefined and beyond the scope of the present review. At present, an ongoing double-blind, multicenter, randomized controlled trial in China is directly evaluating teprotumumab for the treatment of PTM.^40^ The results are anticipated to provide robust evidence to guide future research directions, inform integration into clinical guidelines, and support regulatory approval.

A consistent theme throughout the reviewed literature is the prioritization of functional improvement. Authors repeatedly emphasized mobility, footwear tolerance, pain reduction, and overall quality of life as the most meaningful clinical outcomes, underscoring the disabling nature of PTM. However, in the absence of validated disease-severity instruments, these functional impairments are likely underreported. Future research should formalize this emphasis by developing standardized functional and patient-reported outcome measures, which would allow for more rigorous evaluation of treatment efficacy and facilitate meaningful comparisons across studies.

### Limitations

This review is limited by the predominance of case reports and case series, with only one retrospective study and no controlled trials. Objective PTM outcome measures are lacking, and most studies rely on photographs and patient-reported outcomes, limiting quantitative synthesis. Publication bias toward positive outcomes is highly likely. Case reports describing successful therapeutic responses from an off-label treatment are more likely to be submitted and publishes than cases in which treatment fails or yields minimal benefit. Consequently, the apparent 100% response rate observed in this review certainly overestimates the true effectiveness of teprotumumab for PTM, and clinicians should interpret these findings cautiously until further prospective data from observational studies or randomized controlled trials becomes available. Case-based evidence cannot establish causality, quantify effect size, or define optimal treatment regimens, and spontaneous improvement cannot be excluded entirely. Nonetheless, the consistency of findings across independent reports, coupled with strong mechanistic plausibility, supports further investigation. Given the rarity of PTM, randomized controlled trials may be impractical, highlighting the need for prospective registries and standardized outcome measures. The literature search was limited to PubMed/MEDLINE, CINAHL and Google Scholar; although this meets the general minimum requirement for scoping reviews, additional databases such as Embase, Scopus and Web of Science were not included. Consequently, relevant international case reports indexed exclusively in these databases may not have been captured.

## Conclusion

Teprotumumab is consistently associated with improvement in PTM among patients treated for TED. Integrated clinical and mechanistic evidence supports PTM as an extraocular manifestation of TED with shared IGF-1R–mediated pathophysiology and treatment responsiveness. Prospective registries and standardized outcome measures are needed to define long-term durability and optimal retreatment strategies. Within current evidence limits, these findings remain hypothesis-generating but underscore the importance of studying extrathyroidal manifestations collectively rather than in isolation.

## Disclosure

The authors have no conflicts of interest to disclose.
